# Identification and Expression Analysis of GRAS Transcription Factor Genes Involved in the Control of Arbuscular Mycorrhizal Development in Tomato

**DOI:** 10.3389/fpls.2019.00268

**Published:** 2019-03-15

**Authors:** Tania Ho-Plágaro, Nuria Molinero-Rosales, David Fariña Flores, Miriam Villena Díaz, José Manuel García-Garrido

**Affiliations:** Department of Soil Microbiology and Symbiotic Systems, Estación Experimental del Zaidín (EEZ), CSIC, Granada, Spain

**Keywords:** arbuscular mycorrhiza, tomato, GRAS transcription factors, gibberellins, RNAi interference

## Abstract

The formation and functioning of arbuscular mycorrhizal (AM) symbiosis are complex and tightly regulated processes. Transcriptional regulation mechanisms have been reported to mediate gene expression changes closely associated with arbuscule formation, where nutrients move between the plant and fungus. Numerous genes encoding transcription factors (TFs), with those belonging to the GRAS family being of particular importance, are induced upon mycorrhization. In this study, a screening for candidate transcription factor genes differentially regulated in AM tomato roots showed that more than 30% of known GRAS tomato genes are upregulated upon mycorrhization. Some AM-upregulated GRAS genes were identified as encoding for transcription factors which are putative orthologs of previously identified regulators of mycorrhization in other plant species. The symbiotic role played by other newly identified AM-upregulated GRAS genes remains unknown. Preliminary results on the involvement of tomato *SlGRAS18*, *SlGRAS38*, and *SlGRAS43* from the SCL3, SCL32, and SCR clades, respectively, in mycorrhization are presented. All three showed high transcript levels in the late stages of mycorrhization, and the analysis of promoter activity demonstrated that *SlGRAS18* and *SlGRAS43* are significantly induced in cells containing arbuscules. When *SlGRAS18* and *SlGRAS38* genes were silenced using RNA interference in hairy root composite tomato plants, a delay in mycorrhizal infection was observed, while an increase in mycorrhizal colonization was observed in *SlGRAS43* RNAi roots. The possible mode of action of these TFs during mycorrhization is discussed, with a particular emphasis on the potential involvement of the SHR/SCR/SCL3 module of GRAS TFs in the regulation of gibberellin signaling during mycorrhization, which is analogous to other plant developmental processes.

## Introduction

Arbuscular mycorrhizal (AM) symbiosis occurs between soil fungi and higher plants, including most crop species (Smith and Read, 2008). In this mutualistic association, fungi receive photosynthetically fixed carbohydrates and lipids from plants, whose nutrient acquisition, particularly of phosphates, is improved (Smith and Smith, 2011; Bravo et al., 2017; Jiang et al., 2017; Keymer et al., 2017; Luginbuehl et al., 2017). In addition to nutrient supply, AM symbiosis increases plant resistance and tolerance to biotic and abiotic stress (Augé et al., 2001; Ruiz-Lozano, 2003; Göhre and Paszkowski, 2006; Liu et al., 2007).

AM formation in plant roots is a complex and tightly regulated process requiring signal exchange between plant and AM fungi which activate the symbiosis signaling pathway in host plant roots (Akiyama et al., 2005; Maillet et al., 2011; Genre et al., 2013; Nadal and Paszkowski, 2013; Sun et al., 2015). Although very little is known about the fungal molecular mechanisms controlling the infective process, much greater progress has been made in understanding how the plant orchestrates symbiosis. Cellular reprogramming and transcriptional regulation mechanisms have been reported to accommodate plant roots to arbuscular mycorrhizal symbiosis, particularly in those root cells harboring arbuscules, where nutrient transfers between the plant and fungus occur and are therefore of crucial importance for AM symbiosis (Harrison, 2012; Luginbuehl and Oldroyd, 2017; Pimprikar and Gutjahr, 2018).

To accommodate fungal structures, the host cell undergoes drastic cytological and morphological changes that are accompanied by massive transcriptional reprogramming, which has been observed prior to and during arbuscular formation (Genre et al., 2008; Pimprikar and Gutjahr, 2018). Extensive transcriptional changes are induced during arbuscular formation, and many genes involved in nutrient transport, primary and specialized metabolism, cell wall modification, the secretion pathway, and signal transduction are upregulated in arbuscule-containing cells (Pimprikar and Gutjahr, 2018). In this context, a precise spatiotemporal regulation of gene expression is essential for proper arbuscule development, and the identification of the transcriptional factors mediating these gene expression changes is therefore crucial to understanding how arbuscule formation and function are regulated. At least two coordinated elements regulate arbuscule formation and functioning in root cells: a cell-autonomous wave of gibberellin (GA) accumulation coupled with a GRAS transcription factor network.

GRAS genes, which acronym is based on the first three members to be identified in this family, the gibberellin-acid insensitive (GAI), repressor of GA1 (RGA), and scarecrow-like (SCL) proteins (Pysh et al., 1999), play a crucial regulatory role in a diverse range of fundamental plant biology processes. GRAS protein family has been divided into different subfamilies, each with distinct conserved domains and functions (Tian et al., 2004). GRAS TFs play an important role as regulators of plant development, GA signaling, stress responses, and symbiotic processes (Tian et al., 2004). Several subfamilies of GRAS proteins act as regulators of GA signaling and root development, both of which are important processes that occur during AM formation. DELLA proteins, which share the amino acid sequence DELLA in their N-terminal region, repress gibberellin responses (Silverstone et al., 1998). The SCARECROW (SCR) and SHORT-ROOT (SHR) transcription factors are both involved in radial root organization (Cui et al., 2014), while the SCARECROW-LIKE3 (SCL3) transcription factor, which mediates GA-promoted cell elongation during root development, acts as a coordinator of the GA/DELLA and SCR/SHR pathways (Heo et al., 2011; Zhang et al., 2011).

The DELLA-gibberellin module plays a central role in regulating arbuscule formation (Floss et al., 2013; Foo et al., 2013; Martin-Rodriguez et al., 2016). In a complex with DELLA proteins, CYCLOPS regulates the expression of RAM1 (Pimprikar et al., 2016), which is a GRAS-domain transcription factor capable of interacting with several other GRAS-domain proteins, such as RAD1, and also regulates the expression of genes involved in arbuscule development and nutrient exchanges between the plant and the fungus, including several lipid-biosynthesis and export-related genes such as FatM, RAM2, and STR (Gobbato et al., 2012; Park et al., 2015; Luginbuehl et al., 2017).

Genetic evidence shows that nodulation in legumes as well as mycorrhization in most plant species share a common symbiotic signaling pathway (CSSP), in which GRAS transcription factors, among other actors, play a prominent role. In symbiosis, the first GRAS transcription factor to be characterized was Nodulation Signaling Pathway 2 (NSP2) which forms a DNA binding complex with another GRAS transcription factor, NSP1, and activates early nodulation gene expression (Hirsch et al., 2009). NSP1 and NSP2 genes are also expressed during mycorrhization (Liu et al., 2011), while interaction between NSP2 and RAM1 has also been reported (Gobbato et al., 2012). NSP1 and NSP2, which regulate gene expression related to strigolactone (SL) biosynthesis, can therefore affect the stimulation of mycorrhization mediated by this hormone or other derived compounds such as certain apocarotenoids. Double *nsp1/nsp2* mutants actually have both low SL and mycorrhization levels (Liu et al., 2011).

In recent years, the search for new inducible GRAS TFs during mycorrhization, some of whose symbiotic role has been demonstrated, has been intensified (Xue et al., 2015; Heck et al., 2016; Rich et al., 2017). Thus, the above-mentioned RAM1 is necessary for arbuscule branching and induction of maker genes associated to arbuscule development in *Medicago truncatula*, *Lotus japonicus,* and *Petunia hybrida* (Park et al., 2015; Rich et al., 2015; Xue et al., 2015; Pimprikar et al., 2016), and Required for Arbuscule Development 1 (RAD1) is essential for arbuscular maintenance and functionality (Park et al., 2015). It appears that the relative importance of RAM1 and RAD1 in supporting arbuscule development differs between *Lotus* and *Medicago* and this fact provides an evidence for a diversification in the regulatory networks between plant species (Park et al., 2015; Xue et al., 2015). DELLA Interacting Protein 1 (DIP1), which interacts with RAM1 and DELLA in rice, controls arbuscule formation (Yu et al., 2014), and Mycorrhiza Induced GRAS 1 (MIG1) is necessary for cortex cell shape and size remodeling to accommodate fungal arbuscules (Heck et al., 2016). Given the ability of MIG1 to interact with DELLA1, it has been proposed that a MIG1-DELLA1 complex regulates root development to accommodate fungal infection structures during AM symbiosis. MIG1 belongs to a novel clade of GRAS-domain proteins not present in non-host *Arabidopsis thaliana*, and several members of which are transcriptionally upregulated during mycorrhizal colonization in *M. truncatula*, *L. japonicus*, as well as in *Petunia*, suggesting that this clade of GRAS-domain proteins could play a role in regulating AM development (Xue et al., 2015; Heck et al., 2016; Rich et al., 2017).

As mentioned above, DELLA proteins, a subfamily of GRAS transcription factors, are necessary for the regulation of arbuscule development at different levels, but, paradoxically, are also involved in arbuscule degeneration. Floss et al. (2017) has reported the existence of a transcriptional regulatory complex composed of DELLA and NSP1 which, together with transcription factor MYB1 (a member of the MYB family), forms a regulatory module for the transcription of genes encoding proteins with hydrolytic activities such as proteases and chitinases associated with arbuscular degeneration. Thus, the same set of transcription factors (including DELLA), depending on their combination and association in different regulatory complexes, might modulate both arbuscule formation and arbuscule degeneration.

In this study, we investigated the transcriptional regulation of arbuscule development in tomato roots by screening for candidate GRAS transcription factors. We found that a large number of GRAS genes are induced in mycorrhizal roots, including those previously identified as symbiotic in other plant species. Other genes identified, especially those belonging to the SCL3, SCL32, and SCR subfamilies, had not been studied previously in relation to their possible symbiotic role. We found that AM-induced genes from the SCL3 and SCR subfamilies are expressed in cells containing arbuscules and also regulate AM development. Our results suggest that the GRAS network plays an essential role in transcriptional reprogramming in the tomato host cell during mycorrhization.

## Materials and Methods

### Plant Growth and AM Inoculation

*Solanum lycopersicum* seeds were surface sterilized with 5 min of soaking using 2.35% w/v sodium hypochlorite, subjected to shaking at room temperature for 1 day in the dark, and germinated on a sterilized moistened filter paper for 4 days at 25°C in the dark. Germinated seeds were placed on vermiculite for hypocotyl elongation for 1 week. Each seedling was transferred to a 500-ml pot containing an autoclave-sterilized (20 min at 120°C) mixture of expanded clay, washed vermiculite, and coconut fiber (2:2:1, by volume). In the AM-inoculated (I) treatments, plants were inoculated with a piece of monoxenic culture in Gel-Gro medium produced according to Chabot et al. (1992), containing 50 spores of *R. irregularis* (DAOM 197198) and infected carrot roots. For the non-inoculated (NI) treatment, a piece of Gel-Gro medium containing only uninfected carrot roots was used. Plant growth took place in a growth chamber (day: night cycle; 16 h, 24°C: 8 h, 20°C; relative humidity 50%).

One week after planting and weekly thereafter, the pots were given 20 ml of a modified Long Ashton nutrient solution containing 25% of the standard phosphorus (P) concentration to prevent mycorrhizal inhibition as a result of excess of phosphorous. In the case of non-mycorrhizal plants, the same modified Long Ashton solution was used. Plants were harvested at different times after inoculation. The root system was washed and rinsed several times with tap water, weighed and used for the different measurements according to the nature of the experiments. In each experiment, at least five independent plants were analyzed per treatment.

### Estimation of Root Colonization by AM Fungus

The non-vital trypan blue histochemical staining procedure was used according to the Phillips and Hayman (1970) method. Stained roots were observed with a light microscope, and the intensity of root cortex colonization by AM fungus was determined as described by Trouvelot (1986) using the MYCOCALC software^1^. The parameters measured were frequency of colonization (%F), intensity of colonization (%M), and arbuscular abundance (%A) in the whole root. At least five microscope slides were analyzed per biological replicate, and each slide contained 30 root pieces of 1 cm.

### Plasmid Construction and Hairy Root Transformation

The RNAi fragments of *SlGRAS18* (Solyc01g008910.2.1), *SlGRAS38* (Solyc07g052960.2.1), and *SlGRAS43* (Solyc09g066450.2.1) were amplified from *S. lycopersicum* cDNA of roots infected by the AM fungus *R. irregularis*. The putative promoters of *SlGRAS27* (Solyc02g094340.1), *SlGRAS18*, and *SlGRAS43* (fragments with a size around 1,600 bp immediately upstream of the start codon of the corresponding genes) were obtained from genomic DNA of *S. lycopersicum* cv Moneymaker. Amplifications were carried out by PCR using the iProof High Fidelity DNA-polymerase (BioRad) and specific primers (Supplementary Table 1). PCR fragments were introduced in pENTR/D-TOPO (Invitrogen) vector and sequenced. pENTR/D-TOPO vectors containing the different RNAi or promoter fragments were subsequently recombined into pK7GWIWG2_II-RedRoot^2^ or pBGWFS7::pAtUbq10::DsRed (modified from Karimi et al., 2002) vectors, respectively, using the GATEWAY technology (Invitrogen).

For hairy root transformation, *Agrobacterium rhizogenes* MSU440 cultures harboring the corresponding promoter-GUS and RNAi constructs, were used to transform *S. lycopersicum* cv Moneymaker plantlets according to Ho-Plágaro et al. (2018). Composite plants were transferred to pots and followed the same plant growth conditions as explained before. Screening and selection of DsRed (transformed) roots was done by observation under a fluorescent stereomicroscope Leica M165F.

### Gene Expression Localization

In order to localize the expression of *SlGRAS27, SlGRAS18*, and *SlGRAS43* genes, AM-inoculated and non-inoculated transgenic roots carrying the respective promoter-GUS fusions were used, based on a technique originally developed by Jefferson (1989). Roots transformed by a *PT4* promoter-GUS fusion were used as a control in the same manner as in Ho-Plágaro et al. (2018). Hairy roots carrying the promoter-GUS fusions were vacuum-infiltrated with a GUS staining solution composed of 0.05 M sodium phosphate buffer, 1 mM potassium ferrocyanide, 1 mM potassium ferricyanide, 0.05% Triton X-100, 10.6 mM EDTA-Na, and 5 μg mL-1 X-gluc cyclohexylammonium salt (previously dissolved in N,N-dimethylformamide) for 30 min to improve the penetration of the substrate. Then, the tissues were incubated in the dark at 37°C from 1 h to overnight or until the staining was satisfactory in the same staining solution.

In order to stain the AM fungal structures, the inoculated roots were embedded in 4% agarose blocks and 60-μm transverse sections were cut on a vibratome (Leica VT1200S). Root sections were vacuum-infiltrated with 10 μg ml^−1^ WGA-Alexa Fluor 488 conjugate (Molecular Probes, Eugene, Oreg., USA) in PBS 1X for 60 min in the dark and analyzed under an inverted transmission microscope (Leica DMI600B).

### RNA Extractions and Gene Expression Quantification

For the qPCR experiments, representative root samples from each root system were collected, immediately frozen in liquid nitrogen, and stored at −80°C until RNA extraction. Total RNA was isolated from about 0.2-g samples using the RNeasy Plant Mini Kit (Qiagen, Hilden, Germany) following the manufacturer’s instructions, and treated with RNase-Free DNase. 1 μg of DNAse-treated RNA was reverse-transcribed into cDNA using the iScriptTM cDNA synthesis kit (Bio-Rad, Hercules, CA, USA) following the supplier’s protocol. qPCRs were prepared in a 20-μl reaction volume containing 1 μl of diluted cDNA (1:10), 10 μl 2× SYBR Green Supermix (Bio-Rad, Hercules, CA, USA), and 200 nM of each primer (Supplementary Table 1). A negative control with the RNA sample prior to reverse-transcription was used in order to confirm that the samples were free from DNA contaminations. PCR program consisted of a 3-min incubation at 95°C, followed by 35 cycles of 30 s at 95°C, 30 s at 58–63°C, and 30 s at 72°C. The specificity of the PCR amplification procedure was checked using a melting curve after the final PCR cycle (70 steps of 30 s, from 60 to 95°C, at a heating rate of 0.5°C). Experiments were carried out on three biological replicates, and the threshold cycle (Ct) was determined in triplicate. The relative transcription levels were calculated by using the 2-ΔΔCt method (Livak and Schmittgen, 2001). The Ct values of all genes were normalized to the Ct value of the *LeEF-1α* (accession number X14449) housekeeping gene.

The qPCR data for each gene were shown as relative expression with respect to the control treatment (“reference treatment”) to which it was assigned an expression value of 1. The reference treatment generally corresponded to the non-AM-inoculated treatment. Measurements of transcript abundance of GRAS genes were carried out using the primers previously designed by Huang et al. (2015), and for the qPCR analysis of the reference *LeEF-1α* gene 5′-GGTGGCGAGCATGATTTTGA-3′ and 5′-CGAGCCAACCATGGAAAACAA-3′ primers were used.

### Transcriptome Profiling

Six different root pools (each pool from two different plants) were collected for the RNA-seq analysis: three pools from non-inoculated plants (NI) and three pools from AM-inoculated plants (I). Total RNA was extracted using the Rneasy Plant Mini Kit (Qiagen, Hilden, Germany). cDNA library preparation and sequencing were performed by Sistemas Genómicos S. L. (Paterna, Valencia, Spain) using an Illumina HiSeq1000 machine. The TopHat v2.1.0 algorithm (Trapnell et al., 2009) was used to align reads from the RNA-Seq experiment to the Tomato Genome Reference Sequence SL3.0 provided by the Sol Genomics consortium,^3^ using the last ITAG 3.10 annotation. Then, low-quality reads were removed from the map through Picard Tools,^4^ and high-quality reads were selected for assembly and identification through Bayesian inference using the Cufflinks v2.2.1 algorithm proposed by Trapnell et al. (2010). Gene quantification process was performed by the htseq-count 0.6.1p1 tool (Anders et al., 2015). Isoform quantification and differential expression were carried out through the DESeq2 method (Anders and Huber, 2010). The RNA-seq data have been deposited in the National Center for Biotechnology Information (NCBI) Short Read Archive (SRA) with accession number PRJNA509606.

### Phylogenetic Analysis

Amino acid sequences from the GRAS protein families in tomato (Huang et al., 2015) and *Arabidopsis* (Tian et al., 2004), together with proteins corresponding to AM-related GRAS genes from other plant species, were submitted for the determination of phylogenetic relationships to create a neighbor-joining tree using the Jukes-Cantor model with Geneious software.

### Statistical Analysis

When two means were compared, the data were analyzed using a two-tailed Student’s *t*-test. For comparisons among all means, a one-way or two-way ANOVA was performed followed by the LSD multiple comparison test. The Graphpad Prim version 6.01 (Graphpad Software, San Diego, California, USA) was used to determine statistical significance. Differences at *p* < 0.05 were considered significant. Data represent the mean ± SE.

## Results

### Phylogenetic Analysis of AM-Induced GRAS Transcription Factor Genes in Tomato

**Figure 1:**
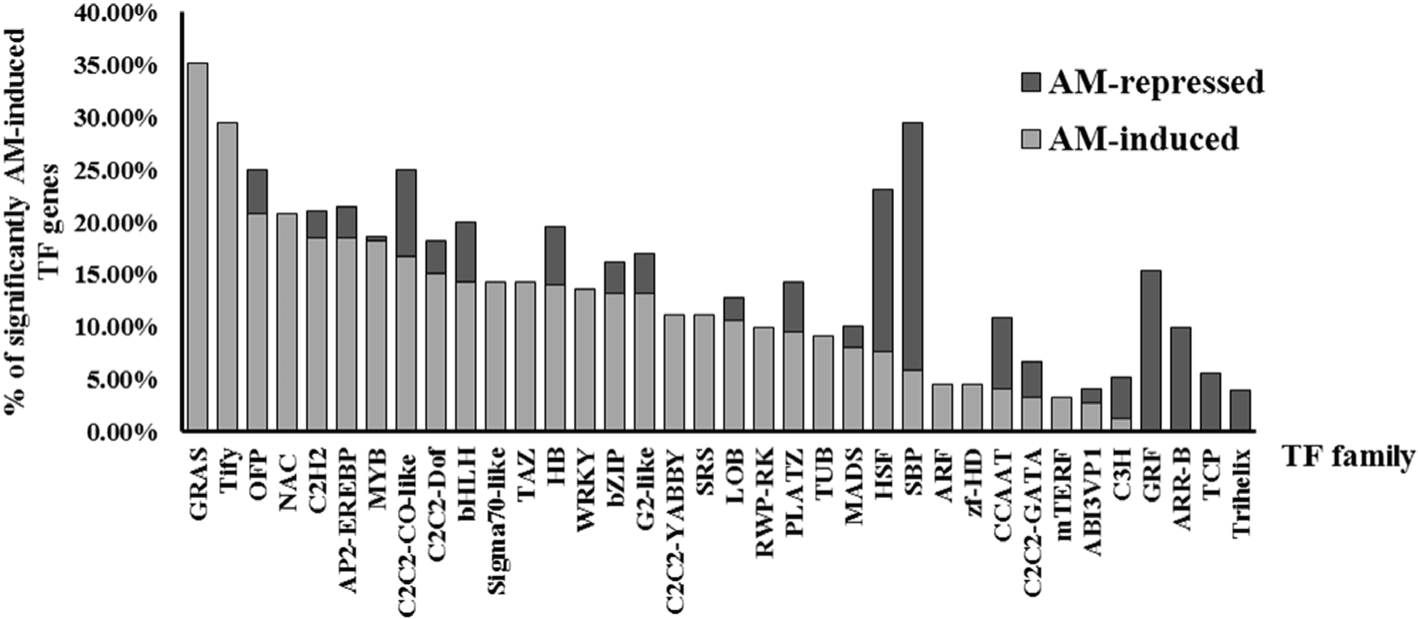
Relative abundance of TF genes differentially regulated upon mycorrhization in tomato. Percentage of AM-repressed and AM-induced TF genes, with RNA-seq fold change <− or > 2 and *p* < 0.05, belonging to each TF gene family in tomato.

To investigate transcriptional regulation of arbuscular mycorrhizal development and to screen for candidate transcription factor genes in tomato, three independent biological replicates of *S. lycopersicum* cv. Moneymaker roots 8 weeks after inoculation with the AM fungus *R. irregularis* (I), exhibiting a 40.2 ± 4% of mycorrhizal colonization, and three non-inoculated (NI) tomato root samples of a similar age were subjected to in-depth messenger RNA sequencing (RNA-seq) analysis. The cutoff for differentially expressed genes (DEGs) was at least a 2-fold induction or repression (*p* < 0.05), and the DEGs coding for transcription factors (TFs) were identified according to the tomato TF list previously published by Geng et al. (2017). As a result, 246 genes encoding transcription factors or transcriptional regulators were significantly upregulated in AM-inoculated roots as compared to non-mycorrhizal roots, while only 59 genes were downregulated (Supplementary Table 2). The most abundant AM-induced TF genes were those encoding the MYB factor family (40 members), APETALA2 and ethylene-responsive element binding protein (AP2-EREBP) family (31 members), C2H2 zinc finger protein family (22 members), NAC protein family (21 members), basic helix–loop–helix (bHLH) protein family (20 members), and the GRAS TF family (19 members). In relative terms, the GRAS transcription factor gene family is the most induced during mycorrhization, followed by the Tify, OFP, and NAC families (Figure 1). Although the downregulation of TF-encoding genes during mycorrhization was not very significant, in some TF families, repression of a large percentage of gene family members, such as SBP (23.53%), GRF, HSF (15.38%), and ARR-B (10%) was observed (Figure 1).

We decided to focus on the GRAS TF gene family. In particular, using RNA-seq analysis, we found that 19 GRAS genes are significantly induced (fold change >2, *p* < 0.05) in AM roots out of a total of 54 tomato GRAS genes, which means that 35.19% of GRAS tomato genes are upregulated upon mycorrhization (Figure 1). Table 1 shows the fold-change induction and corresponding *p* of these 19 AM-upregulated GRAS genes. Similarly, it was recently found that the GRAS TF family was also the most relatively induced during mycorrhization in another *Solanaceae* species, *P. hybrida* (Rich et al., 2017), and in the legume *L. japonicus* (Xue et al., 2015).

**Table 1:** Effect of mycorrhization on the expression of GRAS family genes.

GRAS gene		I *vs* NI	
Huang et al. (2015) nomenclature	SolycDB ID	Fold change	*p*
SlGRAS36	Solyc06g082530	2.023	1.51E-06
SlGRAS46	Solyc10g086530	2.079	2.38E-04
SlGRAS26	Solyc02g092570	4.905	6.22E-10
SlGRAS38	Solyc07g052960	24.058	1.28E-15
SlGRAS39	Solyc08g014030	2.215	6.55E-03
SlGRAS5	Solyc09g018460	2.039	1.03E-02
SlGRAS18	Solyc01g008910	2.012	4.63E-02
SlGRAS47	Solyc11g005610	11.123	1.59E-09
SlGRAS48	Solyc11g013150	2.111	1.99E-04
SlLS	Solyc07g066250	4.383	3.54E-04
SlGRAS37	Solyc07g043330	2.002	2.20E-03
SlGRAS43	Solyc09g066450	49.088	2.15E-37
SlGRAS21	Solyc01g059960	4.006	1.40E-03
SlGRAS22	Solyc01g079370	3.233	2.80E-04
SlGRAS23	Solyc01g079380	2.117	4.54E-02
SlGRAS33	Solyc06g009610	14.588	8.72E-14
SlGRAS27	Solyc02g094340	12.524	6.97E-20
SlGRAS28	Solyc03g110950	35.716	—
SlGRAS49	Solyc11g017100	8.205	—

RNA-seq fold change >2 and *p*-values of GRAS genes upregulated in roots of mycorrhizal plants (I) with respect to non-inoculated (NI) plants.

**Figure 2:**
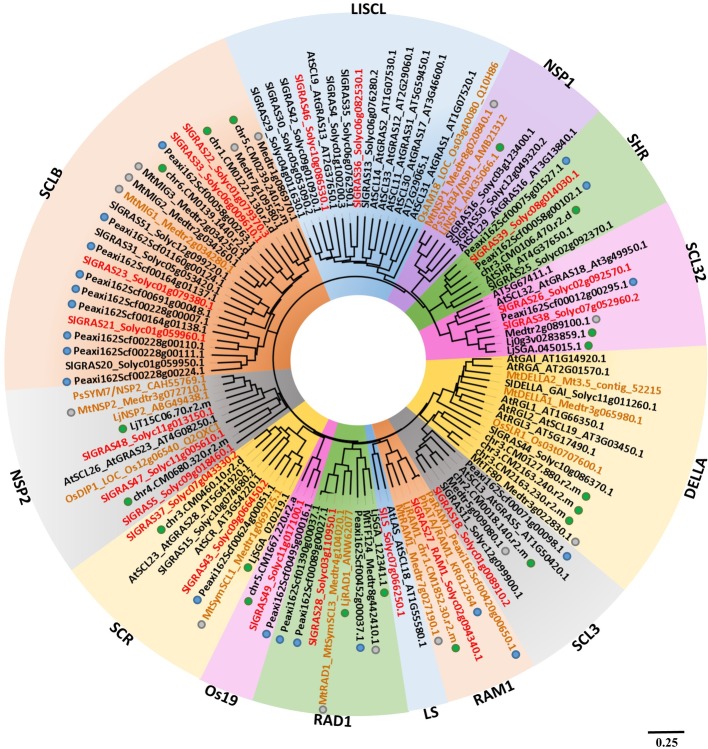
Phylogenetic analysis of AM-related GRAS protein family members in *S. lycopersicum*. An unrooted phylogenetic tree was generated according to amino acid alignment of GRAS proteins from tomato and *Arabidopsis* (Tian et al., 2004), together with AM-induced GRAS members from *P. hybrida* (blue dots), *L. japonicus* (green dots), and *M. truncatula* (gray dots), as well as proteins previously reported to be involved in AM symbiosis (typed in brown) from *L. japonicus* (LjRAD1, LjNSP1, and LjNSP2), *P. hybrida* (PhATA/RAM1), *M. truncatula* (MtDELLA1, MtDELLA2, MtMIG1, MtRAM1, MtNSP1, and MtNSP2), *Oryza sativa* (OsDIP1, OsAM18), and *Pisum sativum* (PsSYM7, PsSYM34). Tomato GRAS proteins, whose corresponding coding genes were induced upon mycorrhization during RNA-seq analysis, are typed in red. Common protein names assigned by Huang et al. (2015) are indicated. The SolDB accessions (http://solgenomics.net/) for tomato proteins and the PlantGDB IDs (http://www.plantgdb.org) for *Arabidopsis* and *Medicago* proteins are also shown. GenBank/RefSeq accession numbers are used for proteins from other species. Background colors correspond to each clade from the GRAS protein family. The scale bar represents the number of substitutions per site.

The GRAS gene family has been previously identified and characterized in tomato (Huang et al., 2015; Niu et al., 2017). We performed a phylogenetic analysis comprising tomato and *Arabidopsis* GRAS proteins, together with other tomato GRAS genes homologous to GRAS members from other species, whose involvement during mycorrhization and/or nodulation has been reported, including DELLA (Floss et al., 2013; Yu et al., 2014; Takeda et al., 2015), RAM1 (Gobbato et al., 2012; Rich et al., 2015; Xue et al., 2015), RAD1 (Xue et al., 2015), and MIG1 genes (Heck et al., 2016). In addition, GRAS genes from *Petunia*, *Lotus*, and *Medicago* previously reported to be AM-induced were also included in the analysis (Xue et al., 2015; Heck et al., 2016; Rich et al., 2017). Figure 2 shows the resulting phylogenetic tree containing the GRAS clades with tomato protein family members corresponding to AM-induced genes, which were designated following the nomenclature described by Huang et al. (2015) and the updated clade classification of Cenci and Rouard (2017). Out of a total of 15 clades of GRAS TFs in tomato, GRAS genes from 11 clades were found to be induced during mycorrhization. Ten clades were coincident with GRAS clades previously reported to contain AM-induced genes in other species (Xue et al., 2015; Heck et al., 2016; Rich et al., 2017), but also a GRAS member from the LS group was firstly detected to be induced during mycorrhization. Among the AM-induced tomato GRAS, we found in tomato some members homologous to genes with a previously established functionality in the mycorrhization process from the following clades: NSP2, RAM1, RAD1, LISCL, and the AM-host exclusive clade SCLB, which includes the MIG1 transcription factor (Maillet et al., 2011; Gobbato et al., 2012; Takeda et al., 2013; Yu et al., 2014; Fiorilli et al., 2015; Xue et al., 2015; Heck et al., 2016; Rich et al., 2017). However, several other tomato GRAS genes induced upon mycorrhization belong to clades, such as Os19, SCL32, LS, SHR, SCR, and SCL3, whose functionality during mycorrhization has not been investigated. Although the function of proteins from the Os19 and SCL32 clades is unknown, LS members are involved in lateral shoot formation during vegetative development (Schumacher et al., 1999; Greb et al., 2003). Finally, SHR, SCR, and SCL3 are involved in a pathway that modulates DELLA/gibberellin signaling involved in root radial patterning and growth, cell division, endodermis specification, and quiescent center identity (Heo et al., 2011; Zhang et al., 2011).

### Tomato Orthologs of GRAS Transcription Factors That Play a Role During Symbiosis

**Figure 3:**
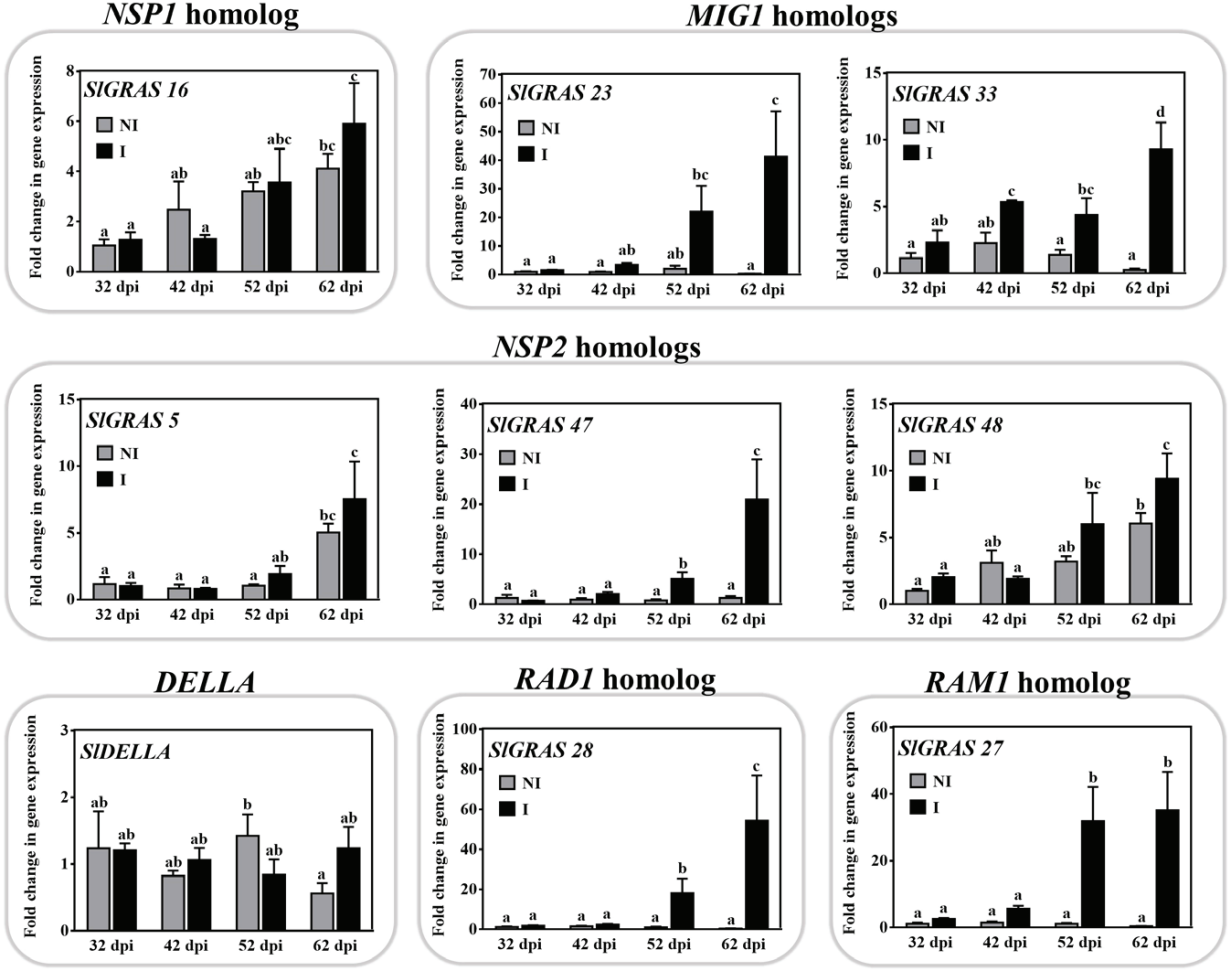
Characterization of expression of mycorrhiza-related GRAS genes in tomato roots. The expression of several putative GRAS genes was analyzed by qPCR in tomato roots after 32, 42, 52, and 62 dpi (days post inoculation) with the AM fungus *R. irregularis*. The qPCR data show relative gene expression in the roots of AM-inoculated plants (I) with respect to their expression in non-inoculated (NI) plants at 32 dpi, with a gene expression of 1. Values correspond to mean ± SE (*n* = 3). Bars with the same letter do not significantly differ (*p* > 0.05) according to the LSD multiple comparison test.

On the basis of the RNA-seq results, we decided to screen the expression of a set of nine tomato GRAS genes, which are putative orthologs known to be involved in mycorrhization. Using qPCR, their expression was assessed in roots in a time-course experiment with *S. lycopersicum* plants after 32, 42, 52, and 62 days post inoculation (dpi) with the AM fungus *R. irregularis* (Figure 3). In these plants, the percentage of mycorrhizal infection gradually increased from 12 ± 0.5% at 35 dpi to 60 ± 5% at 62 dpi. The qPCR data confirmed the mycorrhizal upregulation of *SlGRAS23* and *SlGRAS33* (*MtMIG1* homologs), *SlGRAS47* and *SlGRAS48* genes (close to *LjNSP2*), *SlGRAS28* (*LjRAD1* homolog), and *SlGRAS27* (*MtRAM1* homolog).

The transcripts of *SlGRAS50* and *SlGRAS16*, which are putative orthologs of *NSP1*, did not significantly increase upon mycorrhization (Table 1). However, we hypothesize that the *SlGRAS16* gene might be orthologous to *NSP1*, as the RNA-seq data, with a fold change of 1.96 and *p* < 0.05 (Supplementary Table 2), and the time-course experiment for this gene (Figure 3), indicates a tendency toward gene induction in AM roots.

The AM-induced genes *SlGRAS21*, *SlGRAS22*, *SlGRAS23*, and *SlGRAS33* are putative homologs to the *MtMIG1* gene, which has been reported to be induced in arbuscule-containing cells and to encode a transcription factor suggested as playing a role in cell remodeling during AM fungal accommodation (Heck et al., 2016). In the HAM subfamily, we found that *SlGRAS47, SlGRAS48*, and *SlGRAS5,* according to RNA-seq analysis, are AM-activated genes. *MtNSP2* and the pea gene *PsSYM7,* whose mutation causes a decrease in AM fungal colonization, also belong to this clade (Maillet et al., 2011; Shtark et al., 2016). *SlGRAS28,* whose gene expression has been reported to increase under mycorrhizal conditions, is homologous to *LjRAD1* from *L. japonicus*, whose mutation triggers an acceleration in arbuscule degeneration and a sharp reduction in the number of arbuscules (Xue et al., 2015).

**Figure 4:**
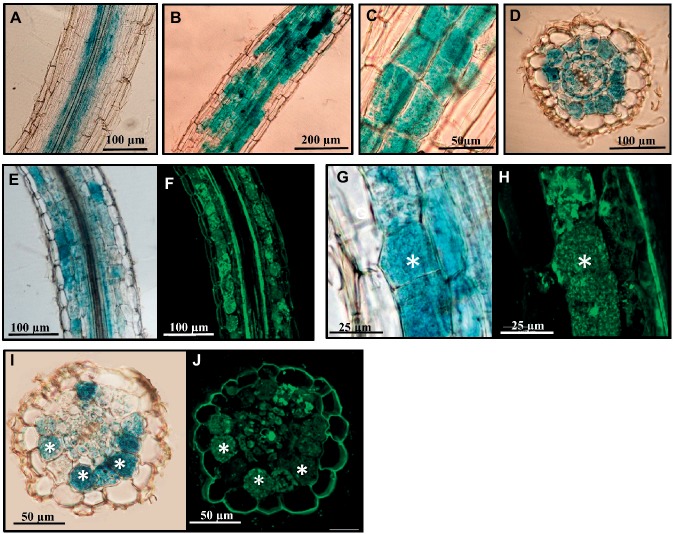
Analysis of the *SlGRAS27* promoter activity in transgenic *S. lycopersicum* roots after GUS staining. GUS activity in sections of *A. rhizogenes*-transformed roots expressing the p*SlGRAS27*:GUS fusion. **(A)** Non-inoculated root. **(B–J)** Transformed roots 8 weeks after inoculation with *R. irregularis*. **(F,H,J)** Images corresponding to root sections from **(E)**, **(G)**, and **(J)**, respectively, co-stained with WGA-Alexa Fluor 488, which labels the AM fungus and fluoresces green. White asterisks indicate arbuscules in the host cortical cells.

*SlGRAS27,* which is also induced upon mycorrhization, is the putative homolog of *MtRAM1* and *PhATA/RAM1* from *Medicago* and *Petunia*, respectively. The localization of the *SlGRAS27* gene’s promoter activity was analyzed. Composite tomato plants with transgenic roots containing the p*SlGRAS27*-GUS fusion were generated, and the promoter of the tomato phosphate transporter (*PT4*), previously reported to be specifically expressed in arbuscule-containing cells (Nagy et al., 2005), was used as a positive control for GUS staining in these cells (Supplementary Figure 1). In non-mycorrhizal roots transformed by the p*SlGRAS27*-GUS fusion, we eventually observed some promoter activity associated with endodermis and pericycle cells (Figure 4A), although, as described by Gobbato et al. (2013) and Park et al. (2015), no staining was detected in most roots. By contrast, under mycorrhizal conditions, promoter activity was observed in AM-colonized cortex zones (Figures 4B–D); this activity appeared to be specifically located in arbuscule-containing cells, as demonstrated by counterstaining with the WGA-alexa488 which enables fluorescent staining of fungal structures (Figures 4E–J).

### Novel AM-Related GRAS Genes From SCL3, SCR, and SCL32 Families

**Figure 5:**
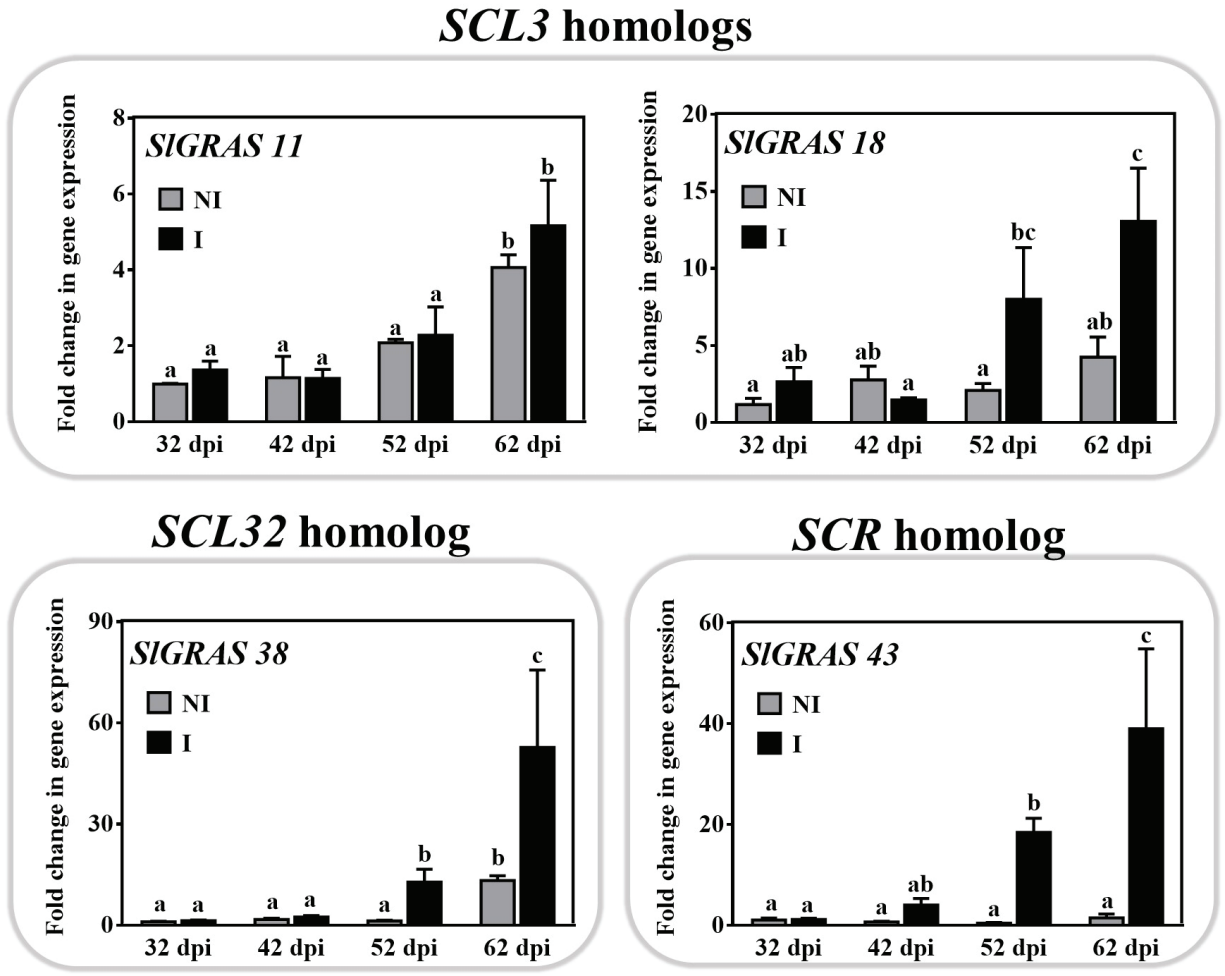
Time-course expression of *GRAS11*, *SlGRAS18*, *SlGRAS38*, and *SlGRAS43* genes during mycorrhization in tomato roots. The expression of putative GRAS genes from SCL3, SCL32, and SCR families, thus far not reported to be related to mycorrhizal symbiosis, was analyzed by qPCR in roots after 32, 42, 52, and 62 dpi (days post inoculation) with the AM fungus *R. irregularis*. The qPCR data represent relative gene expression in the roots of AM-inoculated plants (I) with respect to their expression in non-inoculated (NI) plants at 32 dpi with a designated gene expression of 1. Values correspond to mean ± SE (*n* = 3). Bars with the same letter are not significantly different (*p* > 0.05) according to the LSD multiple comparison test.

Transcripts of the SCARECROW-LIKE3 (SCL3; *SlGRAS18*), SCARECROW-LIKE32 (SCL32; *SlGRAS38*), and SCARECROW (SCR; *SlGRAS43*) families were also induced upon mycorrhization (Figure 2). Their expression increased at the late stages (52–62 dpi) of the interaction (Figure 5) when the roots reached a colonization ratio of 45 ± 3.5% and 60 ± 5%, respectively. Although their putative symbiotic role has not been studied, some members of the SCR, SCL3, and SHR families are known to form a regulatory module that interacts with GA-DELLA signaling in certain root development processes (Heo et al., 2011; Zhang et al., 2011).

**Figure 6:**
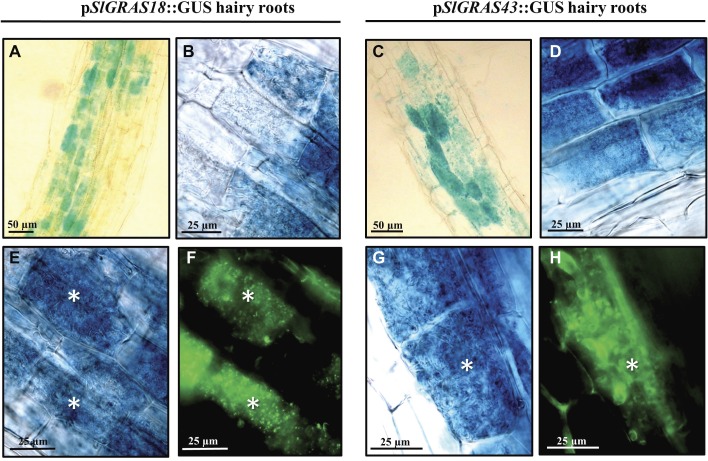
Analysis of the *SlGRAS18* and *SlGRAS43* promoter activity in transgenic *S. lycopersicum* roots after GUS staining. GUS activity in *A. rhizogenes*-transformed roots expressing the p*SlGRAS18*:GUS fusion (images on left) and the p*SlGRAS43*::GUS fusion (images on right) was assessed after 8 weeks of inoculation with *R. irregularis*. **(A–D)** GUS-stained whole roots. **(E,G)** GUS-stained and vibratome-sectioned roots. **(F,H)** Images corresponding to root sections from **(E)** and **(G)**, respectively, co-stained with WGA-Alexa Fluor 488, which labels the AM fungus and fluoresces green. White asterisks indicate arbuscules in the host cortical cells.

The promoter activity of AM-induced genes *SlGRAS18* and *SlGRAS43* was analyzed. A 1600-bp region upstream of each gene was fused to a GUS reporter construct, composite tomato plants were generated and transgenic roots were checked for the presence of the blue dye corresponding to GUS activity. In non-mycorrhizal hairy roots, the promoter activity of the *SlGRAS18* and *SlGRAS43* genes was restricted to cells surrounding the central cylinder and to the apical meristem (Supplementary Figure 2). Although more in-depth analysis is required to determine the specific cell types involved, the expression pattern might be similar to the *Arabidopsis* homologs *SCL3* and *SCR*, described to be induced in the endodermis and the quiescent center (Pysh et al., 1999; Cui et al., 2007; Heo et al., 2011). Mycorrhizal roots examined 8 weeks after inoculation also showed GUS activity in the cortex zones affected by AM fungal root colonization, mainly in arbuscule-containing cells (Figures 6A–D). The GUS-stained roots, which were longitudinally sectioned and counterstained with WGA-Alexa 488 to visualize fungal structures, showed that colonization by *R. irregularis* clearly activated *SlGRAS18* and *SlGRAS43* gene expression in arbusculated cells (Figures 6E–H), although some additional green autofluorescence was also observed around the central stele and in the epidermal root cells.

### RNAi Interference of *SlGRAS18*, *SlGRAS38*, and *SlGRAS43* Genes Affects Mycorrhization

To carry out a functional analysis of *SlGRAS18*, *SlGRAS38*, and *SlGRAS43* genes, RNAi experiments were performed. Composite plants with hairy roots transformed with the control empty vector (pK7GWIWG2_II-RedRoot) and the corresponding RNAi constructs were used. We tested the expression levels of the corresponding silenced GRAS genes at 63 and/or 84 days post inoculation (dpi) and obtained a successful repression of the respective GRAS genes (Supplementary Figure 3), with the exception of the SlGRAS38 RNAi AM-inoculated roots at 84 dpi.

**Figure 7:**
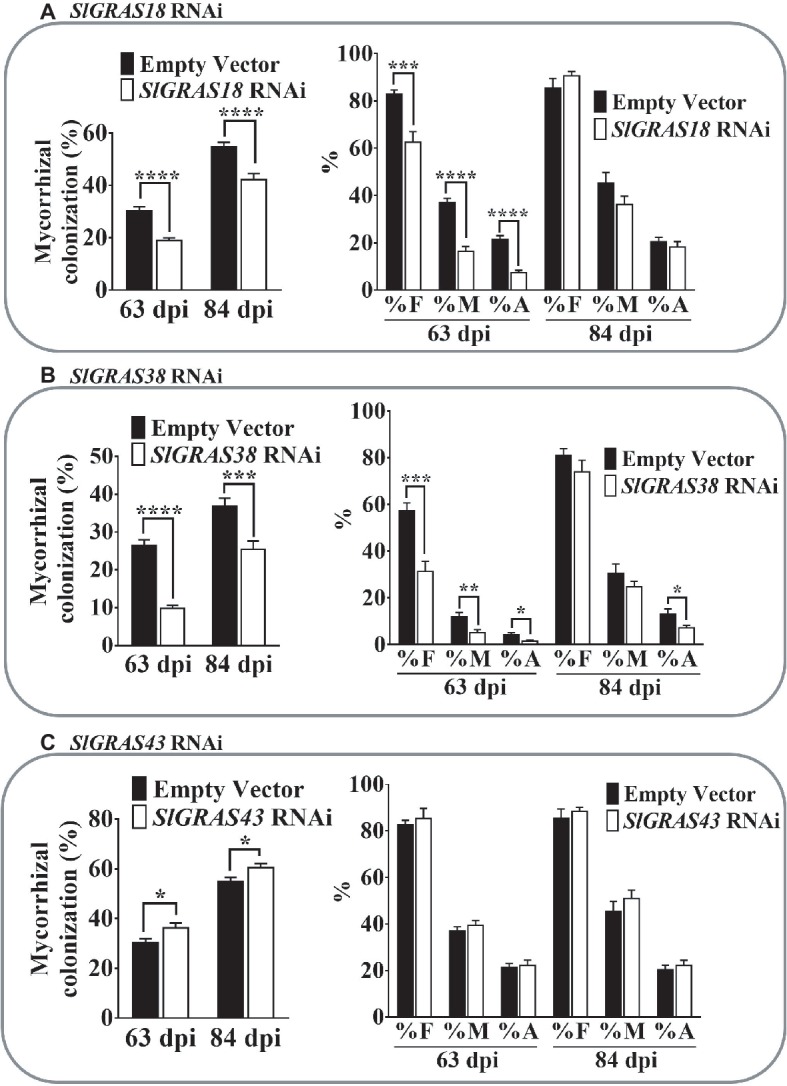
Mycorrhizal colonization parameters in roots of *SlGRAS18*, *SlGRAS38*, and *SlGRAS43* RNAi AM-inoculated composite tomato plants. Percentage of colonized root length (graphs on left) and mycorrhization parameters (graphs on right) were analyzed after 63 and 84 dpi (days post inoculation) with the AM-fungus *R. irregularis* in hairy roots silenced for different GRAS genes. **(A)** *SlGRAS18* RNAi hairy roots. **(B)** *SlGRAS38* RNAi hairy roots. **(C)** *SlGRAS43* RNAi roots. Values correspond to mean ± SE (*n* = 5). Bars with the same letter do not significantly differ. Significant differences (Student’s *t* test) between the mutant and the control are indicated with asterisks (*p < 0.05; **p < 0.01; ***p < 0.001; ****p < 0.0001).

Analysis of the percentage of mycorrhization in the different *GRAS* RNAi roots revealed important changes. Compared to the control, *SlGRAS18* RNAi roots showed a delay in the colonization process, with a significant decrease in the percentage of root length colonized by the AM fungus observed at both harvesting times (63 and 84 dpi) (Figure 7A). A significant decrease in all the mycorrhizal parameters measured was observed at 63 dpi, but not at 84 dpi (Figure 7A), indicating that *SlGRAS18* gene silencing negatively affects relative mycorrhizal intensity (M%), as well as arbuscule abundance (A%), and that these differences disappear at late stages of mycorrhization.

Similar results were obtained for the *SlGRAS38* RNAi roots. A significant reduction in mycorrhizal colonization was also observed and, again, F, M, and A% were negatively affected in *SlGRAS38*-silenced roots at 63 dpi, although only arbuscular intensity remained significantly reduced at 84 dpi (Figure 7B).

*SlGRAS43* RNAi composite plants presented a significant increase in the percentage of root length colonized by the AM fungus, but no significant differences in the mycorrhizal parameters were observed (Figure 7C).

## Discussion

Transcriptional reprograming of root cellular development in AM symbiosis is being intensely studied. However, although our understanding of the molecular mechanisms involved has increased considerably in recent years (Pimprikar and Gutjahr, 2018), only some components of the transcriptional machinery and certain cis-regulatory elements essential for AM-specific gene expression in arbuscule-containing cells have been described (Rubio et al., 2001; Karandashov et al., 2004; Chen et al., 2011). In this study, RNA-seq analysis revealed a more than two-fold induction (*p* < 0.05) of 246 putative transcription factors during tomato mycorrhization (Supplementary Table 2), with the GRAS family showing the highest relative abundance level of AM-induced TF genes. A total of 19 GRAS genes were identified as upregulated upon mycorrhization. As for *Petunia* (Rich et al., 2017) and *Lotus* (Xue et al., 2015), the GRAS genes were prominent among the AM-inducible TF genes in tomato. In addition, some of the AM-induced GRAS genes, particularly those belonging to the SCLB, RAD1, RAM1 clades, did not cluster with any of the genes in the non-mycorrhizal plant *Arabidopsis.* These facts reinforce the idea that GRAS TFs are conserved core components involved in transcriptional reprogramming in plant roots required for AM formation.

Although the functionality of most GRAS gene family members remains undetermined, several have been functionally characterized and found to have diverse functions and to play an important role in many plant growth and development processes (Bolle, 2015), probably due, to a great extent, to their ability to regulate gibberellic acid (GA) responses (Sun, 2010; Zhang et al., 2011; Gong et al., 2016).

Several GRAS gene family members, including DELLA (Floss et al., 2013; Foo et al., 2013; Yu et al., 2014; Takeda et al., 2015), RAM1 (Gobbato et al., 2012; Rich et al., 2015; Xue et al., 2015), RAD1 (Xue et al., 2015), and MIG1 (Heck et al., 2016), have been reported to play a major role during mycorrhization and nodulation. The RNA-seq analysis found an overall alteration in the expression of genes encoding GRAS transcription factors, thus suggesting that the GRAS network plays an essential role in transcriptional reprogramming in the tomato host cell during mycorrhization, as reported for other species (Gobbato et al., 2012; Rich et al., 2015; Xue et al., 2015). In this study, we identified the symbiotic *RAM1, NSP2, RAD1, MIG1*, and *DIP1* GRAS genes, whose expression patterns corroborate their AM inducibility. In our experiments, although *SlGRAS16*, a putative ortholog of NSP1, did not increase significantly upon mycorrhization, a tendency toward gene induction in AM roots was observed in the RNA-seq and qPCR data. Thus, as with its homologs MtNSP1, OsNSP1, LjNSP1, and PsSYM34/NSP1 in other species, SlGRAS16 could be expected to have a mycorrhizal-related function. The mutation or silencing of *NSP1* gives rise to an impaired response to Myc-LCOs at the pre-symbiotic stage and reduced frequency of mycorrhizal colonization in *Medicago* (Delaux et al., 2013; Hohnjec et al., 2015), delayed AM development in pea (Shtark et al., 2016), and a decrease in the frequency of *L. japonicus* plant colonization by AM fungi (Takeda et al., 2013).

We found that *SlGRAS27* is the putative homolog of *PhATA/RAM1* and that *SlGRAS27* promoter expression appeared to be specifically located in arbuscule-containing cells. Likewise, Park et al. (2015) showed that the promoter expression of *MtRAM1*, the homolog of *SlGRAS27* in *M. truncatula*, appears to be associated with arbuscule-containing cells. This finding differs from that of Gobbato et al. (2013), who reported that the *MtRAM1* promoter presents homogenous activity in all AM-colonized root tissues, rather than specifically in cortical cells harboring arbuscules. These divergent observations suggest that the specific localization of *RAM1* expression during mycorrhization may depend on the plant species and mycorrhizal stage.

In this study, several tomato GRAS genes from the SCL32, SHR, SCR, and SCL3 clades were found to be induced in mycorrhizal roots. Although putative orthologs of these members have been functionally analyzed in the non-host *Arabidopsis*, their putative symbiotic role has not yet been studied in AM- host species or nodulating plants. Finely tuned hormonal signaling, in which the gibberellin/DELLA module plays an essential role, is well known to occur during mycorrhization (Floss et al., 2013; Takeda et al., 2015). SCL3 and the upstream transcription factors SHR and SCR are involved in gibberellin response regulation. SCL3, in particular, which acts as a positive regulator of gibberellin signaling, antagonistically interacts with DELLA, and is transcriptionally activated *via* SHR-SCR in certain root development processes (Heo et al., 2011; Zhang et al., 2011). In this respect, we hypothesized that the SHR/SCR/SCL3 module of GRAS TFs may act as a regulator during mycorrhization by modulating GA signaling.

In order to investigate the possible role played by SHR, SCR, and SCL3 GRAS transcription factors during mycorrhization, we focused on tomato putative orthologs which exhibit higher expression transcript levels under mycorrhizal conditions and selected the *SlGRAS18*, *SlGRAS43*, and S*lGRAS38* genes for further analysis. *SlGRAS18* was the only GRAS member from the SCL3 clade to show some gene induction upon mycorrhization. Of the three tomato GRAS proteins belonging to the SHR clade, *SlGRAS43* was undoubtedly the most AM-upregulated gene, which showed a 49-fold induction in our RNA-seq analysis. Finally, although *SlGRAS39* is the putative AM-induced ortholog gene of *AtSHR* (Figure 2), instead, we decided to analyze *SlGRAS38*, belonging to the closely related SCL32 clade, which showed a 24-fold induction in AM-roots as compared to a two-fold induction for *SlGRAS39*. We functionally characterized these three tomato GRAS genes during mycorrhization using RNA interference. All the experiments were performed with transformed composite tomato plants, which is a useful technique as a first screening and a less time-consuming approach than the whole plant transformation (Ho-Plágaro et al., 2018) which will be required to test the exact role of the genes in mycorrhizal functioning. The observed effects on the mycorrhizal parameters due to RNA interference were more evident for *SlGRAS18* and *SlGRAS38* at the first harvest, where roots showed a lower level of all the measured parameters, indicating that the transgenic RNAi effect was probably not effective enough at late stages with higher levels of mycorrhization in roots.

Our phylogenetic analysis suggests that SlGRAS18 could be a tomato ortholog of AtSCL3. AtSCL3 and DELLA are reported to interact and antagonize each other in regulating GA biosynthetic genes and GA responses. AtSCL3 thus attenuates DELLA in the root endodermis, acts as a positive regulator of GA signaling, and integrates GA/DELLA signaling and the SHR/SCR pathway into root elongation (Zhang et al., 2011). In this study, we show that the putative tomato homolog *SlGRAS18,* whose gene expression is higher in the late stages of mycorrhization when arbuscules are abundant in the root and whose silencing triggers a delay in mycorrhizal colonization and a decrease in mycorrhizal parameters, is expressed in arbuscule-containing cells. Given these results, we hypothesize that the *SlGRAS18* gene, which, like AtSCL3, is able to positively regulate GA signaling antagonizing DELLA (Li et al., 2011), could be involved in controlling the action of GAs in arbuscular mycorrhiza development. Gibberellin signaling exerts positive and negative effects on arbuscular mycorrhiza development by interfering with symbiotic signaling and gene expression (Takeda et al., 2015). Thus, the decrease observed in arbuscule content, colonization frequency, and intensity of mycorrhization in *SlGRAS18* RNAi roots may occur by an increase in arbuscule degradation mediated by DELLA in mature arbuscules (Floss et al., 2017) or by a reduced hyphal branching and development in root mediated by low GA conditions (Takeda et al., 2015).

*SlGRAS38* RNAi was found to have a similar effect on mycorrhizal colonization patterns as *SlGRAS18* silencing, thus suggesting the possibility of a signaling pathway *via SlGRAS38(SHR)—SlGRAS18(SCL3)* which controls arbuscular mycorrhiza development through the induction of GA signaling and is analogous to the action of the SHR-SCR-SCL3 module for GA signaling induction in *Arabidopsis* (Heo et al., 2011; Zhang et al., 2011). SlGRAS38 belongs to the functionally uncharacterized SCL32 clade (Cenci and Rouard, 2017), which is phylogenetically close to the SHR and NSP1 proteins. In previous studies, all the SCL32, SHR, and NSP1 proteins were considered to belong to the SHR clade (Huang et al., 2015). In addition, given the close phylogenetic proximity of SlGRAS38 to NSP1, its direct role in arbuscule degeneration through the formation of a complex composed of DELLA and MYB1, as reported by Floss et al. (2017) for NSP1 in *M. truncatula,* cannot be ruled out.

Finally, our study shows that *SlGRAS43*, which is strongly induced in the arbusculated cortex cells of AM roots, belongs to the SCR clade. Its *Arabidopsis* homologs, AtSCR and AtSCL23, interact with each other and are needed to specify endodermal cell fate in the root meristem (Long et al., 2015) and bundle sheath cell fate in leaves (Cui et al., 2014). Although their role in symbiotic root interactions is poorly studied, curiously, *MtSymSCL1* (Medtr1g069725.1), the closest *SlGRAS43* homolog in *M. truncatula,* is induced in both mycorrhizal and nodulated roots (GEA database) and its silencing triggers a decrease in the number of nodules (Kim and Nam, 2013). An opposite result was observed in *SlGRAS43* RNAi hairy roots, which showed a slight increase in mycorrhizal colonization. After analyzing mycorrhizal parameters, no significant differences were observed. The alterations in mycorrhizal patterns upon *SlGRAS43* silencing need to be studied in more depth in order to obtain a clearer picture of the possible regulating role of *SlGRAS43* during mycorrhization.

In light of the above-mentioned hypothesis, the *SlGRAS18* and *SlGRAS38* genes could be involved as members of the hypothetical SHR-SCR-SCL3 module in a coordinated regulation of arbuscule formation/degeneration during mycorrhization by regulating DELLA repressor of GA signaling. However, our preliminary results indicate that *SlGRAS15* or the AM-induced *SlGRAS37,* rather than *SlGRAS43,* may be potential candidate orthologs of *SCR* in tomato.

In conclusion, our results indicate that, in addition to some classic GRAS transcription factors involved in AM symbiosis, other members of the GRAS family, such as SCL3, SCR, and SCL32, are involved in regulating the mycorrhizal process in tomato. The data provided in this study, together with previous functional analyses of the respective genes, suggest that gibberellin signaling during mycorrhizal symbiosis could be regulated by these other transcription factors from the GRAS family in conjunction with members of the well-known DELLA gene subfamily.

## Author Contributions

JG-G and TH-P designed the experiments. NM-R, DF, and MD performed the experiments. NM-R, TH-P, and JG-G analyzed the data. TH-P and JG-G edited the manuscript.

### Conflict of Interest Statement

The authors declare that the research was conducted in the absence of any commercial or financial relationships that could be construed as a potential conflict of interest.

The handling editor declared a shared affiliation, though no other collaboration, with the authors at time of review.

## Footnotes

^1^http://www.dijon.inra.fr/mychintec/Mycocalc-prg/download.html

^2^http://gateway.psb.ugent.be

^3^https://solgenomics.net/organism/solanumlycopersicum/genome

^4^http://picard.sourceforge.net

## Abbreviations

AM: Arbuscular Mycorrhiza
GA: Gibberellins
qPCR: Quantitative RT-PCR
SHR: SHORT-ROOT
SCR: SCARECROW
SCL: SCARECROW-LIKE.
