# Supplementary material for: Identification and Expression Analysis of GRAS Transcription Factor Genes Involved in the Control of Arbuscular Mycorrhizal Development in Tomato
Source: Front Plant Sci. 2019 Mar 15;10:268. doi: 10.3389/fpls.2019.00268 (PMC6429219; doi:10.3389/fpls.2019.00268)
Supplement: Supplementary file 1 [file Data_Sheet_1.PDF]

**Supplementary Table 1. Primers used in this study for PCR amplifications and plasmid constructions**

| Target sequence | Primer name | Primer sequence (5´→3´) |
|---|---|---|
| ***SlGRAS18* RNAi** | iGRAS18-F | (5´-CACCGTTCATTTGCAAAGGGCAAT -3´) |
| | iGRAS18-R | (5´-TGCAACAATCTCCTTGCTTG -3´) |
| ***SlGRAS38* RNAi** | iGRAS38-F | (5´-CACCTTGGGAGCCTTGATGGAGC -3´) |
| | iGRAS38-R | (5´- GCAATGGAGGAACTTGTGGT-3´) |
| ***SlGRAS43* RNAi** | iGRAS43-F | (5´-CACCGGACCCTCTTGTGCTGAAAG -3´) |
| | iGRAS43-R | (5´-TTGCACCCAATGTATGGCTA -3´) |
| ***SlGRAS27* promoter** | promRAM1-F | (5´- CACCGGTAGCAGGAGGACTTGTAC -3´) |
| | promRAM1-R | (5´-GTTACCACACACCTGCTTTTTG-3´) |
| ***SlGRAS18* promoter** | promGRAS18-F | (5´-CACCGTCCTTAAATAGAGAGTG -3´) |
| | promGRAS18-R | (5´-TGTGGTGAATTCTTGATATC -3´) |
| ***SlGRAS43* promoter** | promGRAS43 -F | (5´- CACCGAGGACGAGTCAGAAG -3´) |
| | promGRAS43 -R | (5´- TGAGTAGATGAGGAGAATTGAGTT-3´) |

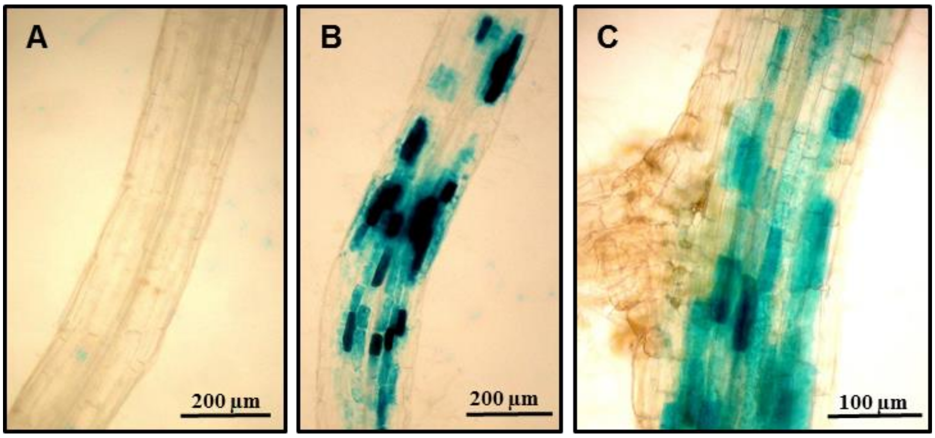


**Supplementary Figure 1. Expression analysis of the *SlPT4* promoter in transgenic *S. lycopersicum* roots after GUS staining.** GUS activity in *A. rhizogenes* transformed roots expressing the p*SlPT4*:GUS fusion. (A) Non-inoculated root. (B-C) Transformed roots eight weeks after inoculation with *R. irregularis*.

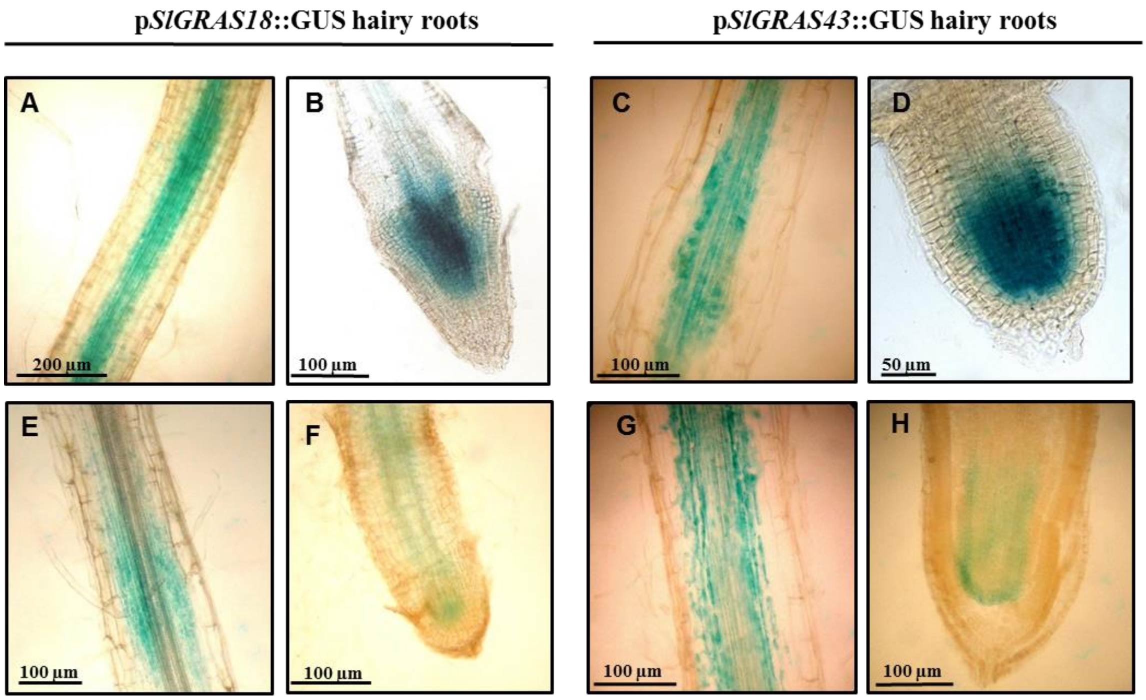


**Supplementary Figure 2. Expression analysis of the *SlGRAS18* and *SlGRAS43* promoters in transgenic *S. lycopersicum* non-mycorrhizal roots after GUS staining.** GUS activity in *A. rhizogenes* transformed roots expressing the p*SlGRAS18*:GUS (images on the left) and the p*SlGRAS43*::GUS (on the right) fusions was assessed. (A-D) Whole root. (E-H) Longitudinal sections performed with a vibratome.

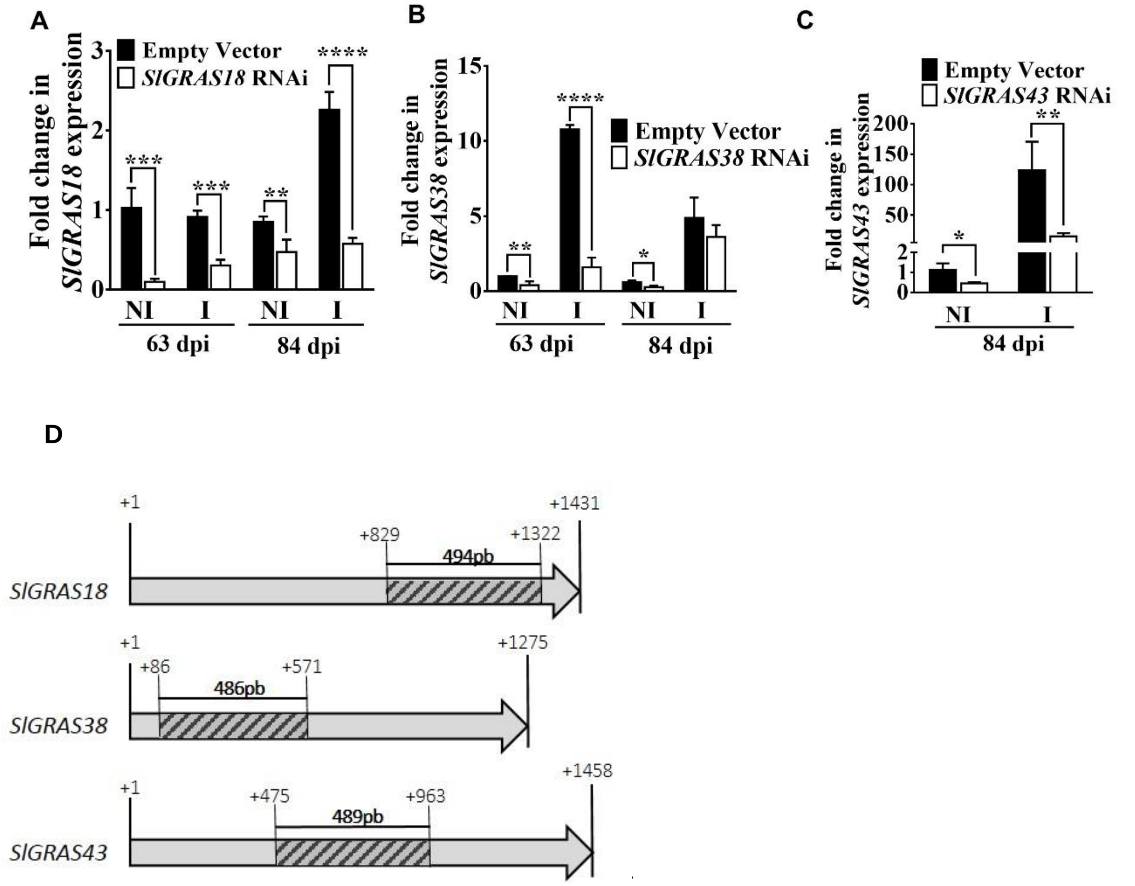


**Supplementary Figure 3. *SlGRAS18, SlGRAS38* and *SlGRAS43* gene silencing in RNAi tomato hairy roots.** The transcript abundance of the corresponding silenced genes was quantified by qPCR in hairy roots from RNAi composite plants non-inoculated (NI) and inoculated (I) with the AM fungus *R. irregularis* at 63 and 84 dpi. (A) *SlGRAS18* RNAi roots. (B) *SlGRAS38* RNAi roots. (C) *SlGRAS43* RNAi roots. (D) DNA fragments for RNAi silencing of each gene (dashed background), where the base pairs inside the coding sequence are numbered. Significant differences (Student´s t test) between the mutant and the control are indicated with asterisks (*P<0.05; **P<0.01; ***P<0.001;****P<0.0001).
